# Supplementary material for: ATP biosensor reveals microbial energetic dynamics and facilitates bioproduction
Source: Nat Commun. 2024 Jun 21;15:5299. doi: 10.1038/s41467-024-49579-1 (PMC11192931; doi:10.1038/s41467-024-49579-1)
Supplement: Supplementary file 1 — Supplementary Information [file 41467_2024_49579_MOESM1_ESM.pdf]

Supplementary Information for

ATP biosensor reveals microbial energetic dynamics and facilitates bioproduction

Authors: Xinyue Mu[a], Trent D. Evans[a], Fuzhong Zhang[a-c*]

[a]Department of Energy, Environmental and Chemical Engineering

[b]Division of Biological & Biomedical Sciences

[c]Institute of Materials Science & Engineering

Washington University in St. Louis, Saint Louis, MO 63130, USA

[*] **Correspondence:**

Dr. Fuzhong Zhang

1 Brookings Drive, Campus Box 1180, St. Louis, MO, 63130, USA

Email: fzhang@seas.wustl.edu

Table of Contents

Supplementary Table 1..1
Supplementary Table 2..1
Supplementary Figure 1..2
Supplementary Figure 2..3
Supplementary Figure 3..4
Supplementary Figure 4..5
Supplementary Figure 5..6
Supplementary Figure 6..7
Supplementary Figure 7..8
Supplementary Figure 8..9
Supplementary Figure 9..10
Supplementary Figure 10..11
Supplementary Figure 11..12
Supplementary Figure 12..13
Supplementary note...14
Supplementary Reference..17

Supplementary Table 1. Strains used in this study

Strains	Relevant genotype	refs
***E. coli* MG1655**	F^- λ^- ilvG- rfb-50 rph-1	
***E. coli* NCM3722**	F^+	
***E. coli* DH1**	F^- endA1 recA1 gyrA96 thi-1 glnV44 relA1 hsdR17(rK-mK+) λ^-	
***E. coli* DH1ΔfadE**	F^- endA1 recA1 gyrA96 thi-1 glnV44 relA1 hsdR17(rK-mK+) λ^- ΔfadE	[1]
***P. putida* KT2440**	rmo- mod+	
***P. putida* KT2440 ΔphaJ4**	rmo- mod+ ΔphaJ4	this study
**sXM01**	*E. coli* DH1ΔfadE: pA5c-tesA, pSJ23119a-CFP-iATPsnFR1.1	this study
**sXM02**	*E. coli* DH1: pS6k-mCherry-iATPsnFR1.1	this study
**sXM03**	*E. coli* MG1655: pS6k-mCherry-iATPsnFR1.1	this study
**sXM04**	*P. putida* KT2440: pB2k-mCherry-iATPsnFR1.1	this study
**sXM05**	*P. putida* KT2440 ΔphaJ4: pB2k-mCherry-iATPsnFR1.1	this study
**sXM06**	*P. putida* KT2440: pTrc-phaC1J4, pB6g-mCherry-iATPsnFR1.1	this study
**sXM07**	*E. coli* DH1: pJBEI-6409, pS6a-CFP-iATPsnFR1.1	this study
**sXM08**	*E. coli* DH1: pJBEI-6409-M1M2, pSJ23119a-CFP-iATPsnFR1.1	this study
**sXM09**	*E. coli* DH1: pJBEI-6409-M2M3, pSJ23119a-CFP-iATPsnFR1.1	this study
**sXM10**	*E. coli* DH1: pJBEI-6409-M1M3, pSJ23119a-CFP-iATPsnFR1.1	this study
**sXM11**	*E. coli* DH1: pJBEI-6409-M1M2, pJBEI-6409-M3, pSJ23119a-CFP-iATPsnFR1.1	this study
**sXM12**	*E. coli* DH1: pJBEI-6409-ΔatoB, pSJ23119a-CFP-iATPsnFR1.1	this study
**sTE01**	*E. coli* NCM3722: pS6k-mCherry-iATPsnFR1.1	this study

Supplementary Table 2. Plasmid used in this study

Plasmids	replication origin	promoter	resistance	description	refs
**pA5c-tesA**	p15A	PlacUV5	Cm^R	FA production	[1]
**pS6k-mCherry-iATPsnFR1.1**	pSC101	PLlacO1	Kan^R	Inducible ATP sensor for *E. coli*	this study
**pB6k-mCherry-iATPsnFR1.1**	BBR1	PLlacO1	Kan^R	Inducible ATP sensor for *P. putida*	this study
**pB6g-mCherry-iATPsnFR1.1**	BBR1	PLlacO1	Gen^R		this study
**pSJ23119a-CFP-iATPsnFR1.1**	pSC101	PJ23119	Amp^R	Constitutive ATP sensor for *E. coli*	this study
**pJBEI-6409**	p15A	PlacUV5, Ptrc	Cm^R	Limonene pathway	[2]
**pTac-phaC1J4**	pVS1, p15A	Ptac	Kan^R	PHA production	[3]
**pJBEI-6409-ΔatoB**	p15A	PlacUV5, Ptrc	Cm^R	Partial limonene pathway containing different modules	this study
**pJBEI-6409-M1M2**	p15A	PlacUV5, Ptrc	Cm^R		this study
**pJBEI-6409-M2M3**	p15A	PlacUV5, Ptrc	Cm^R		this study
**pJBEI-6409-M1M3**	p15A	PlacUV5, Ptrc	Cm^R		this study
**pB8k-6409-M3**	BBR1	pBAD	Kan^R		this study
**pCas9**	pSEVA224	PJ23105	Gen^R	phaJ4 deletion	[4]
**pJOE_phaJ4**	BR322	Plac	Kan^R		this study
**pgRNAtet-phaJ4**	BBR1	PJ23119	Tet^R		this study

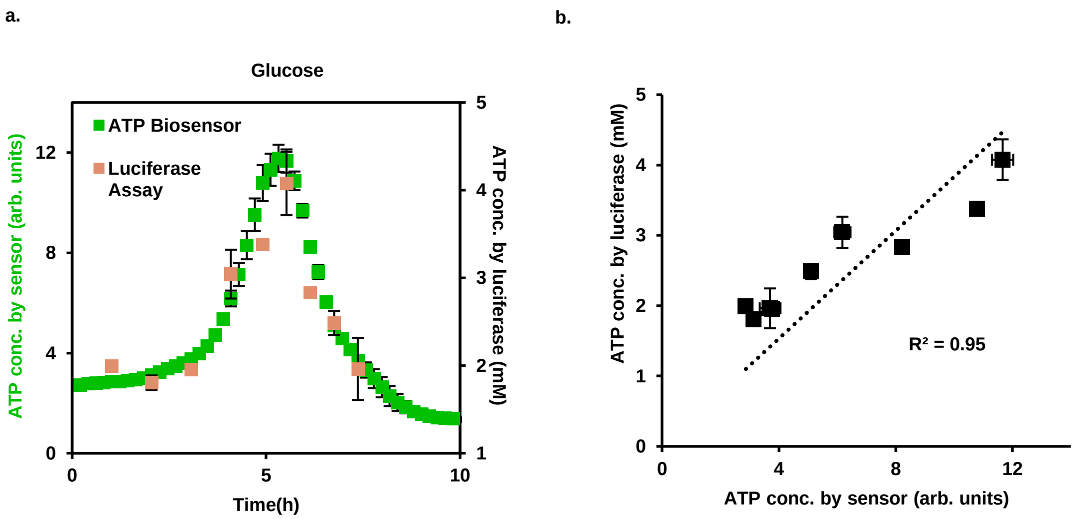

Supplementary Figure 1 (a) *Escherichia coli* ATP dynamics in M9 media with 0.4% glucose, measured by the biosensor (green square) and the luciferase assay (orange square), respectively. (b) Correlation of ATP concentration measured by the ATP biosensor and the luciferase assay. Error bars represent standard deviation from 3 biological replicates (n=3).

a.

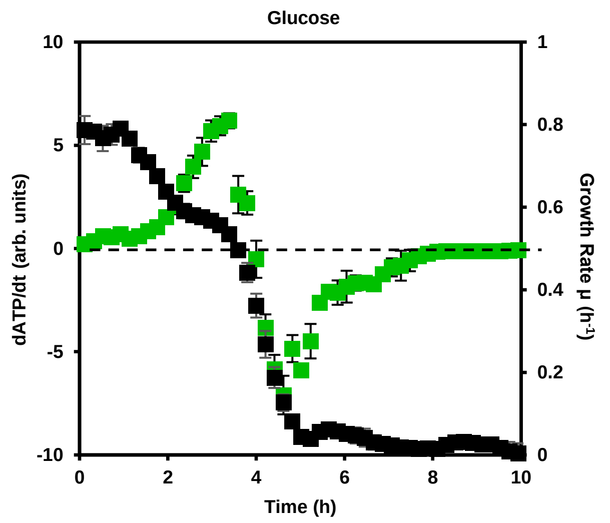

b.

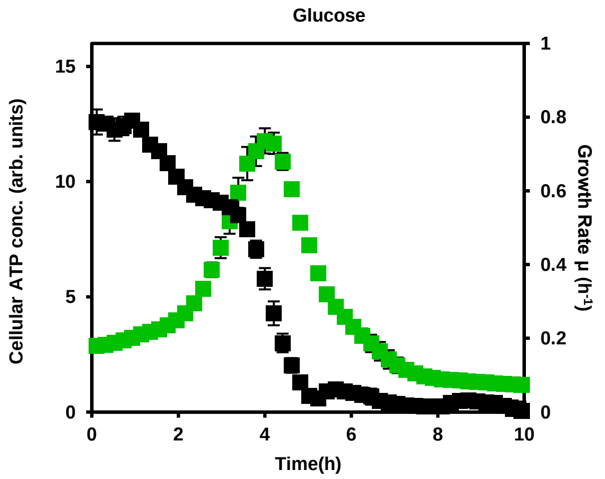

Supplementary Figure 2 (a) The rate of ATP concentration change (dATP/dt) and instantaneous growth rate in M9 media with 0.4% glucose. (b) *E. coli* cellular ATP concentration and instantaneous growth rate in M9 media with 0.4% glucose. Error bars represent standard deviation from 3 biological replicates (n=3).

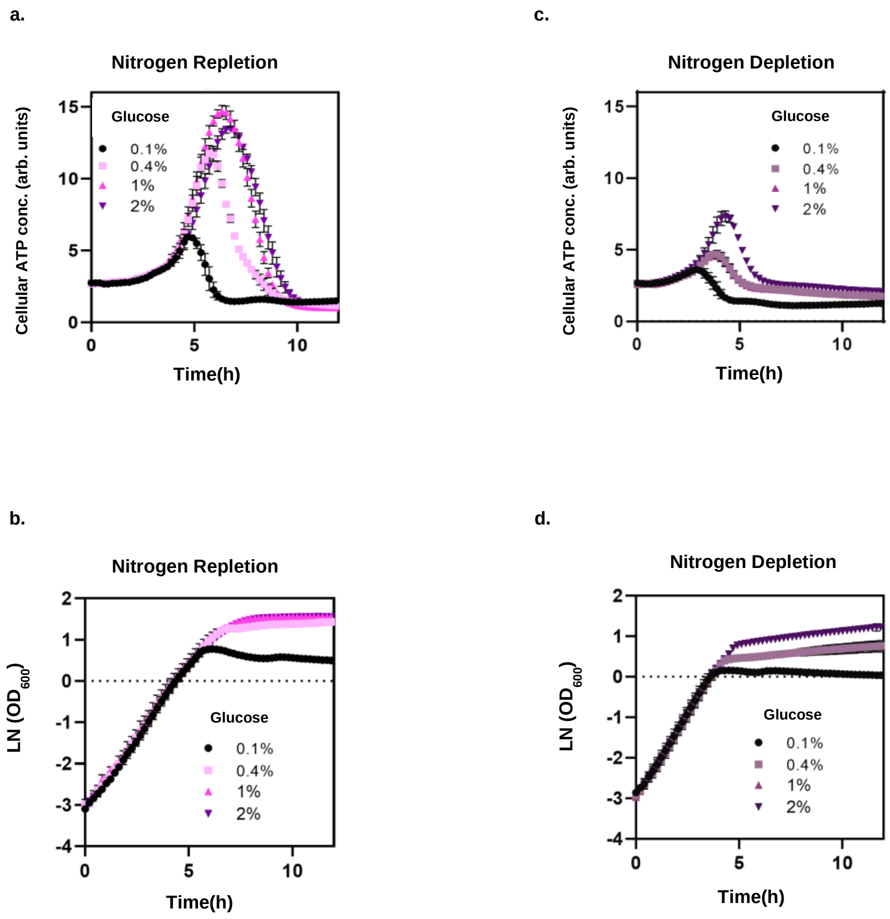

Supplementary Figure 3 (a) *E. coli* ATP dynamics and (b) cell growth in M9 media with different amount of glucose under nitrogen repletion condition. (c) ATP dynamics and (d) cell growth in M9 media with different amount of glucose under nitrogen limited condition. Error bars represent standard deviation from 3 biological replicates (n=3).

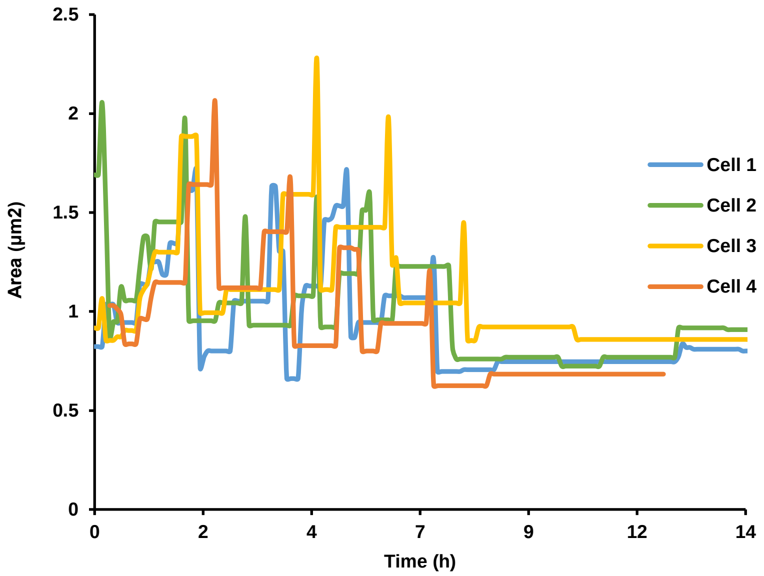

Supplementary Figure 4 Area change of representative *E. coli* cells growing on an agarose pad supplemented with M9 glucose (0.1%) medium. Cell areas were quantified from time-lapse video as shown in Supplementary Video.

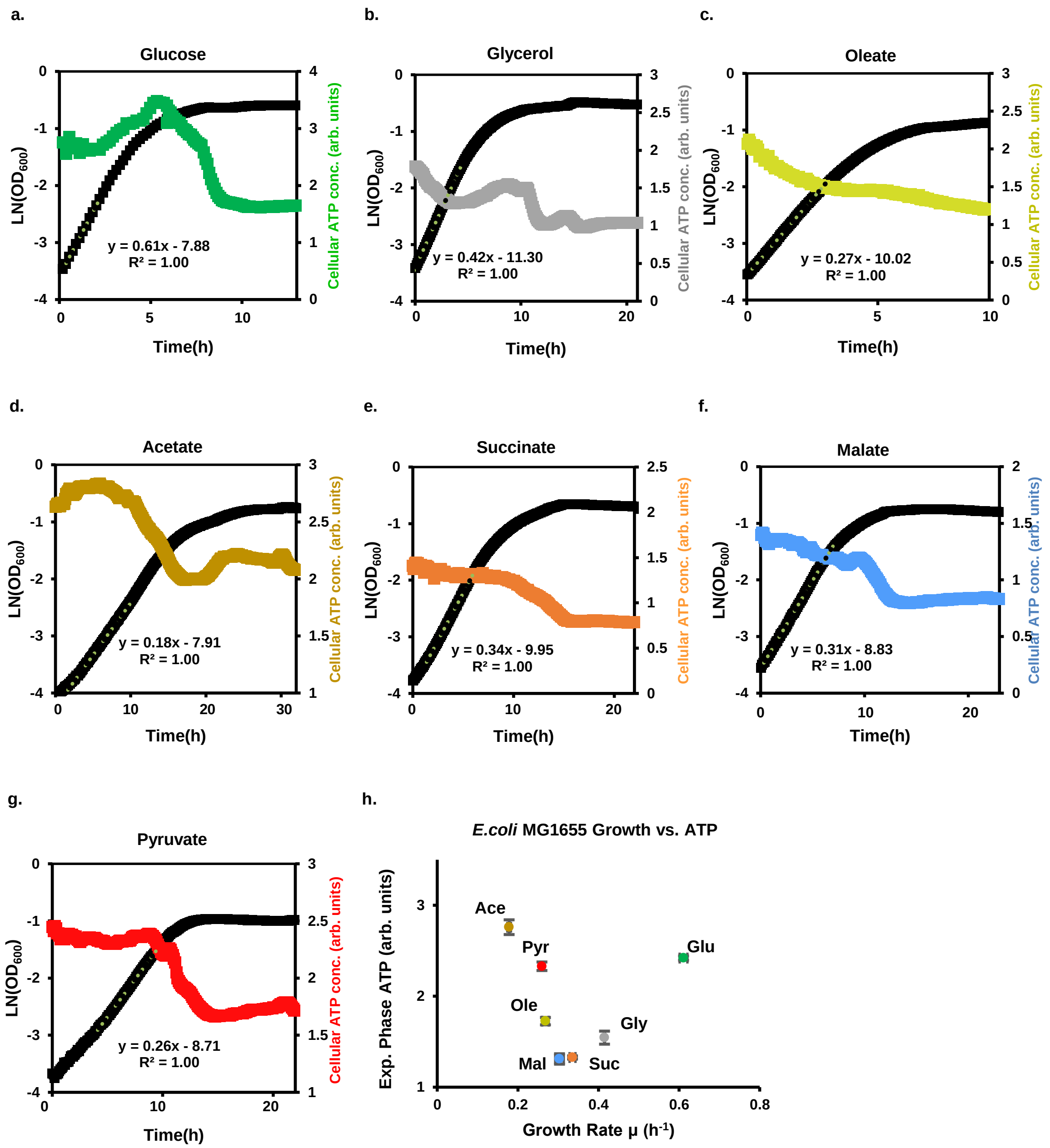

Supplementary Figure 5 ATP dynamics of *E. coli* MG1655 using different carbon sources. (a-g) ATP dynamics and OD of *E. coli* MG1655 using glucose, glycerol, oleate, acetate, succinate, malate and pyruvate. (h) Growth vs. steady-state ATP. Error bars represent standard deviation from 3 biological replicates (n=3).

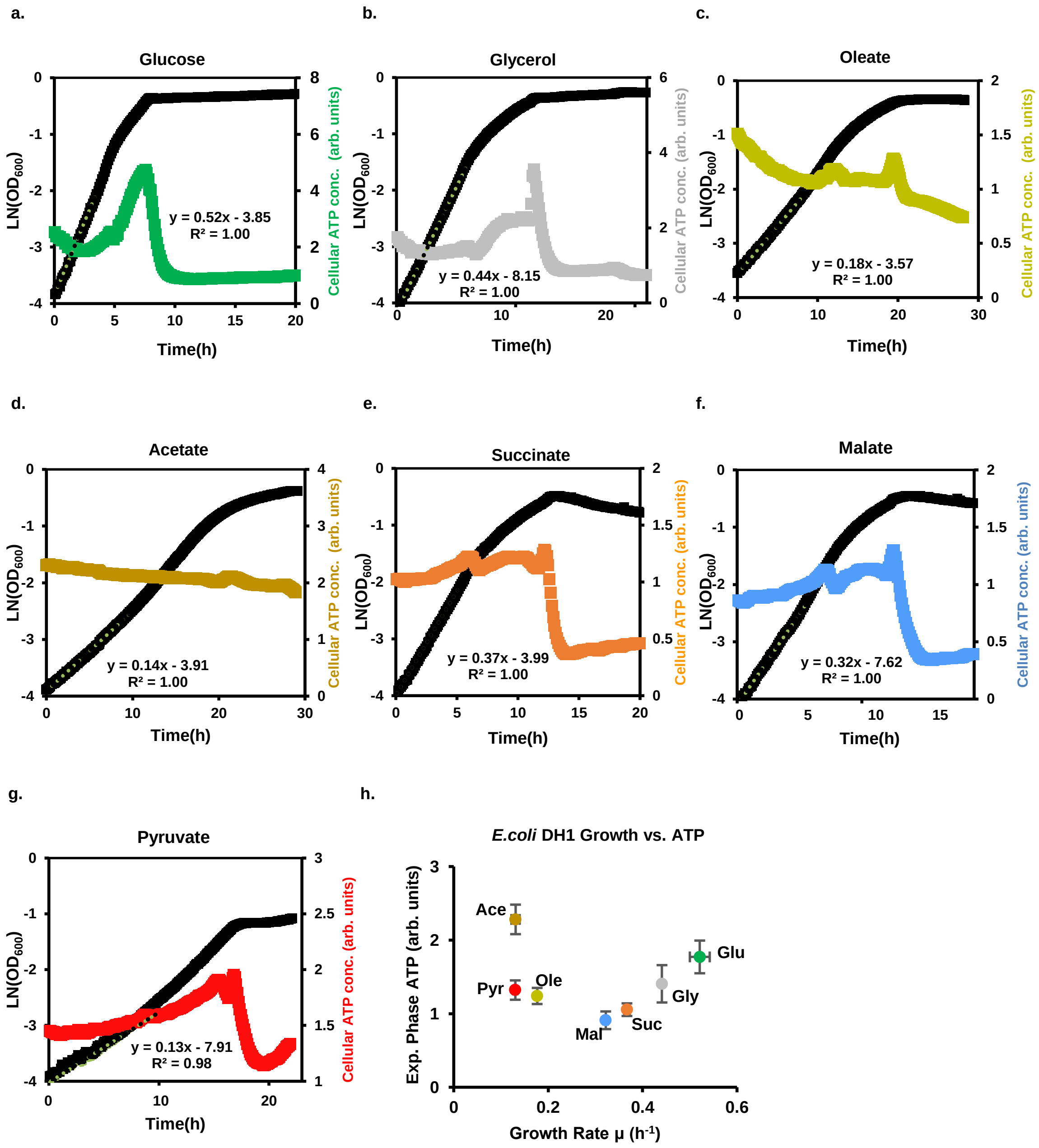

Supplementary Figure 6 ATP dynamics of *E. coli* DH1 using different carbon sources. (a-g) ATP dynamics and OD of *E. coli* DH1 using glucose, glycerol, oleate, acetate, succinate, malate and pyruvate. (h) Growth vs. steady-state ATP. Error bars represent standard deviation from 3 biological replicates (n=3).

a.

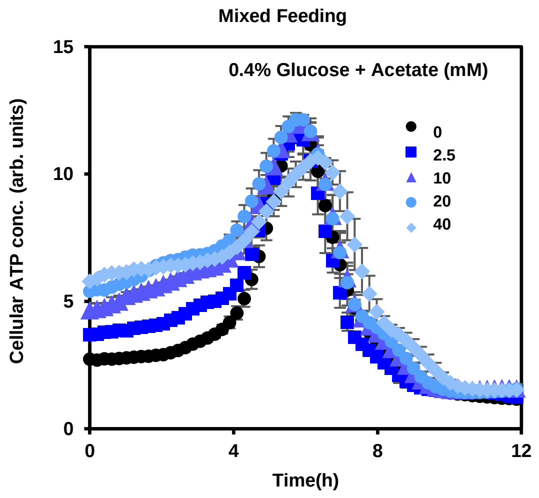

b.

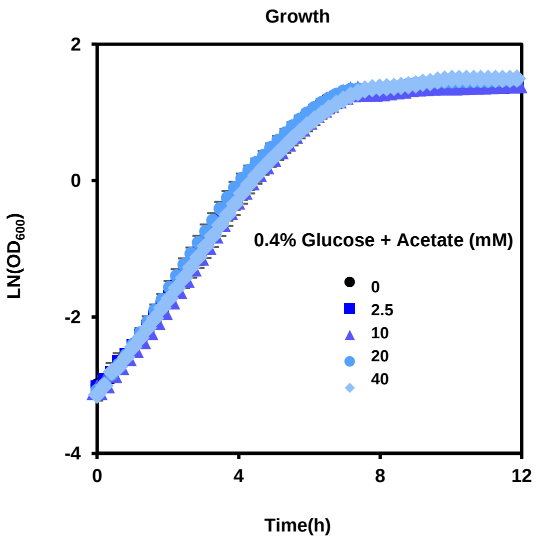

Supplementary Figure 7 (a) ATP dynamics and (b) cell growth in mixed feeding media with 0.4% glucose and different amounts of acetate. Error bars represent standard deviation from 3 biological replicates (n=3).

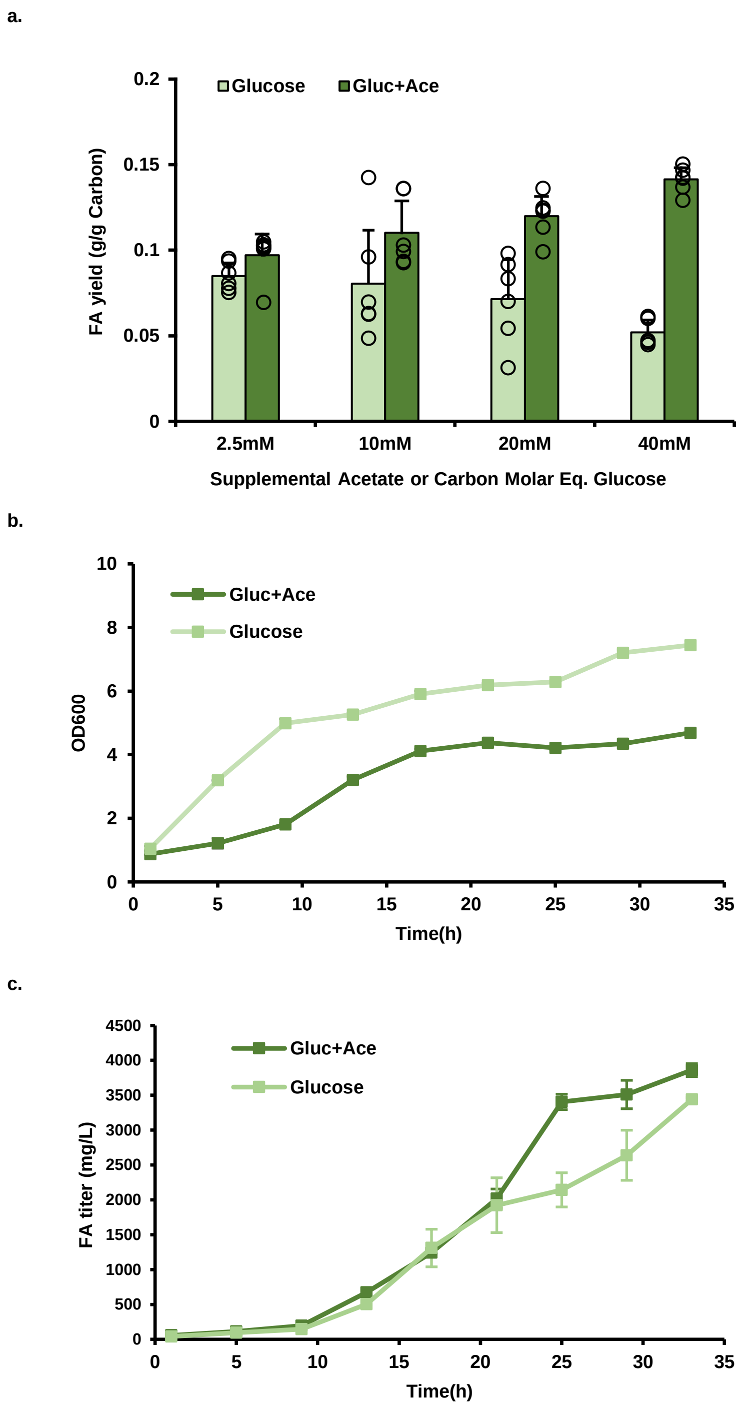

Supplementary Figure 8 FA production in cultures with 40 mM additional carbon. (a) FA yield. Error bars represent standard deviation from 6 biological replicates (n=6). (b) Growth curves. (c) FA titers over time. Error bars represent standard deviation from 3 biological replicates (n=3).

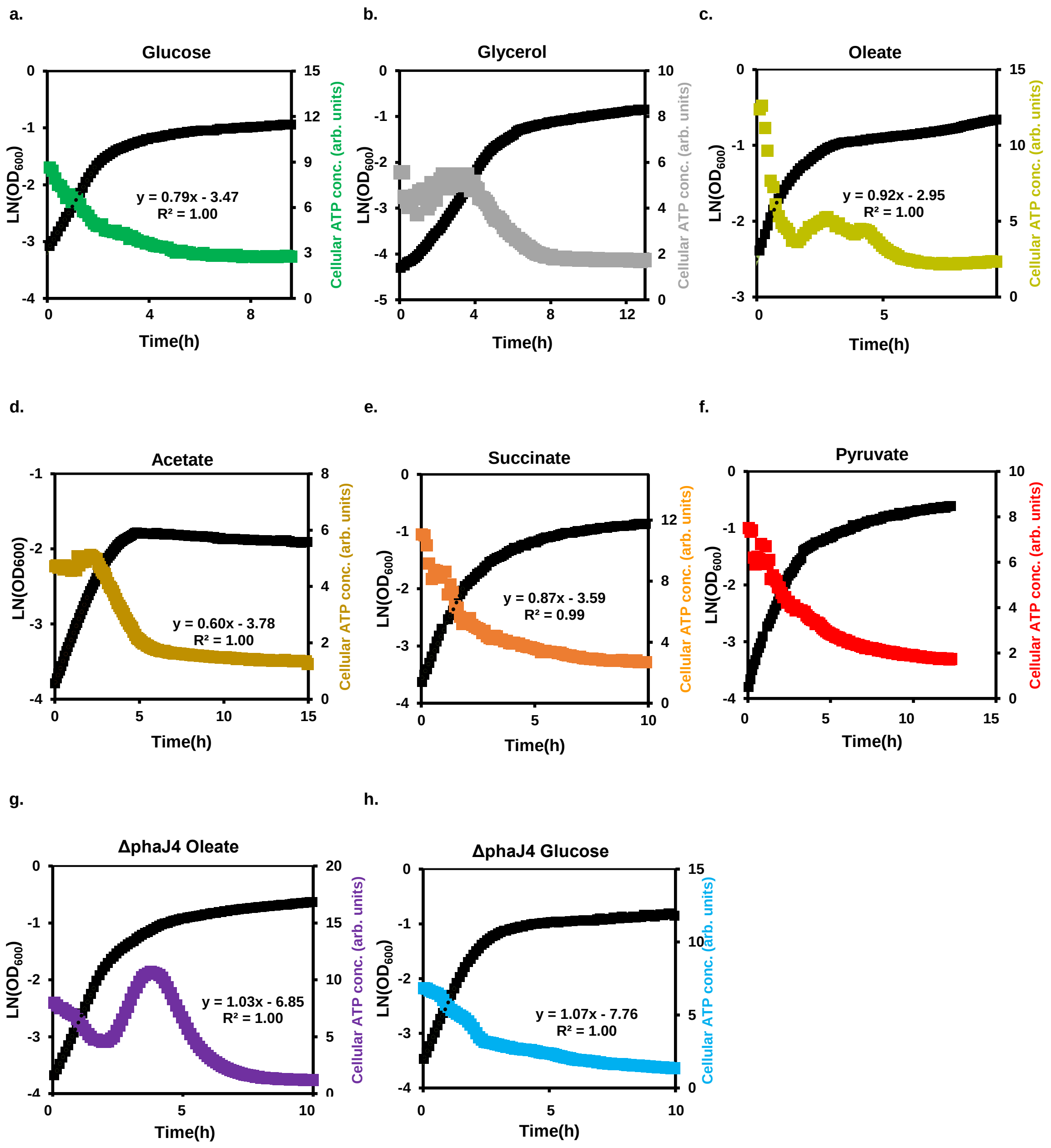

Supplementary Figure 9 ATP dynamics of *P. putida* KT2440 using different carbon sources. (a-f) ATP dynamics and OD of *P. putida* KT2440 growing in minimal glucose, glycerol, oleate, succinate, pyruvate, and acetate. (g-h) ATP dynamics and OD of *P. putida* ΔphaJ4 using glucose and oleate. Error bars represent standard deviation from 3 biological replicates (n=3).

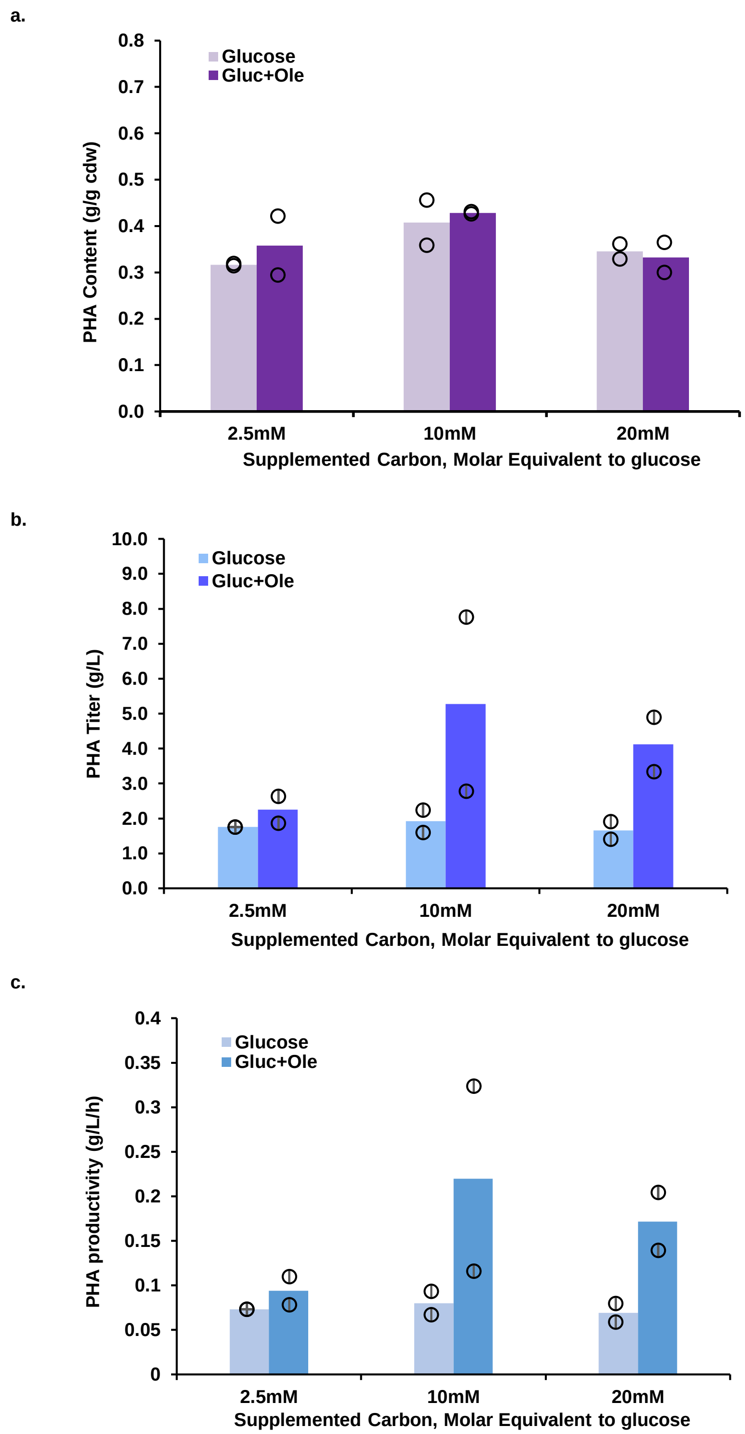

Supplementary Figure 10 (a) PHA content of *P. putida* ΔphaJ4 after 24 h fermentation in different amounts of glucose or oleate supplementation. (b) PHA titer and (c) productivity of the wild-type *P. putida* after 24h fermentation in additional amounts of glucose or oleate supplementation. Data were obtained from 2 biological replicates (n=2).

a.

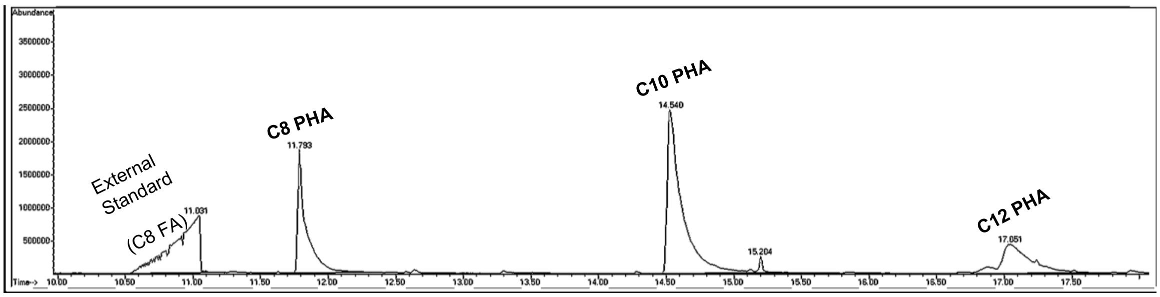

b.

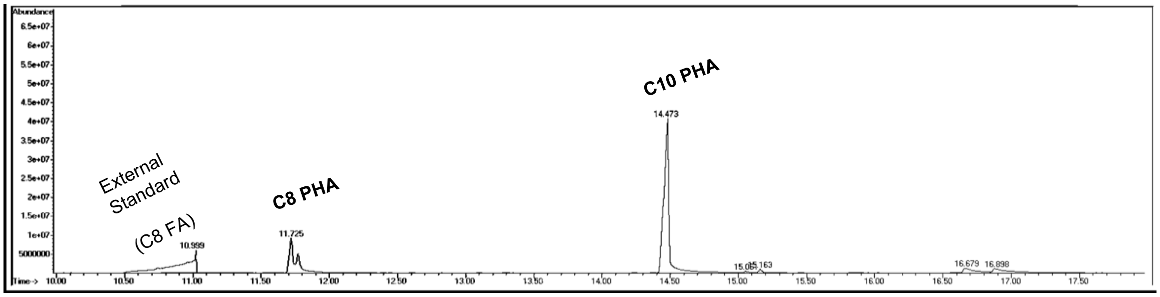

Supplementary Figure 11 PHA profiles of (a) the wildtype and (b) ΔphaJ4 *P. putida* strain.

a.

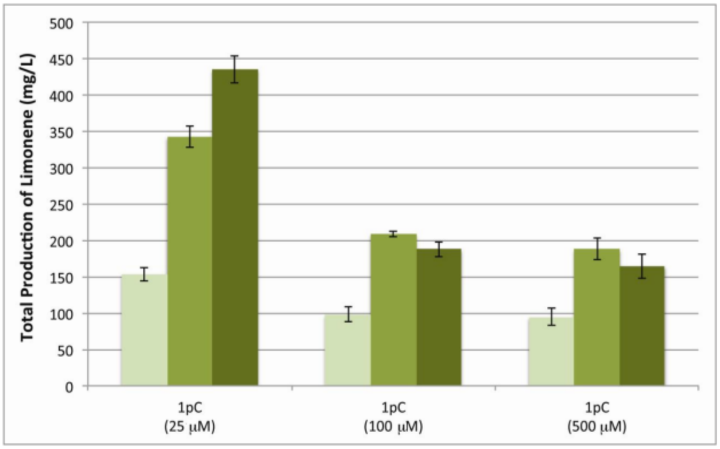

b.

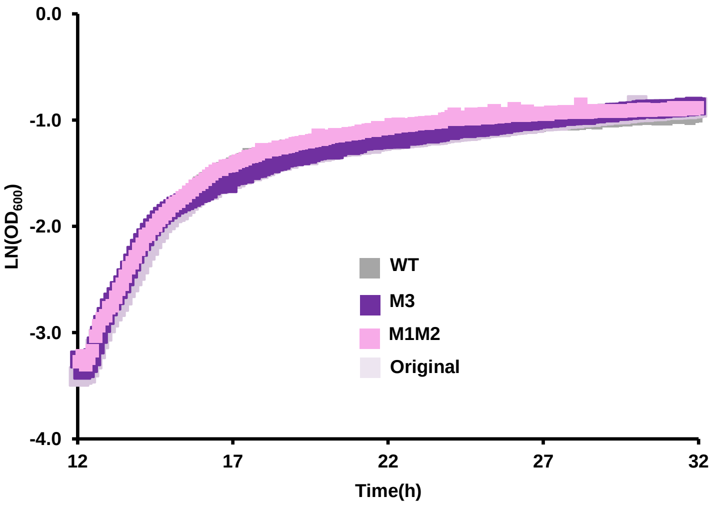

Supplementary Figure 12 (a) Limonene titers at different induction levels after 24 hours of fermentation.[2] Reprinted from *Metabolic Engineering*, **19**, Alonso-Gutierrez, J. et al., Metabolic engineering of Escherichia coli for limonene and perillyl alcohol production, 33-41, Copyright (2013), with permission from Elsevier. Supplementary Figure 2 in the original publication [2] (b) Growth of *E. coli* strains expressing the original limonene pathway or partial limonene pathways. Error bars represent standard deviation from 3 biological replicates (n=3).

Supplementary Note

Modeling steady state ATP concentration.

To understand the intracellular ATP concentration [ATP] across different carbon sources, we consider both ATP production rate $r_{prodction}$ and consumption rate $r_{consumption}$:

$$\frac{d[ATP]}{dt} = r_{prodction} - r_{consumption} \tag{1}$$

The ATP production rate, with the unit of M/s, can be calculated from ATP production coefficient k_{prod} (in the unit of ATP moles per gram dry cell weight per hour), which can be further calculated from carbon metabolism:

$$r_{prodction} = k_{prod}\frac{M(\mu)}{V(\mu)} \tag{2}$$

where M represents cell mass, and V represents cell volume. Both M and V are growth-rate dependent. In *E. coli*, the relationships between steady state M and V with µ have been modeled using empirical equations[5]:

$$M = -0.636 + 0.635 \cdot 2^{0.718\mu} \tag{3}$$

$$V = 0.486 \cdot 2^{1.144\mu} \tag{4}$$

For wild type bacteria cells, ATP is mostly consumed for cell growth and maintenance, which can be modeled by zero and first order kinetics, respectively [6]:

$$r_{consumption} = k_g\mu + k_m[ATP] \tag{5}$$

where k_g is the ATP consumption coefficient by cell growth. The $k_g\mu$ term represents the combine ATP-consuming reactions needed for cell growth. k_m is the first-order maintenance energy consumption rate coefficient. ATP consumption during carbon metabolism, such as activation of glucose to G6P will be considered later when calculating the ATP production coefficients. Combining equations (1), (2), and (5):

$$\frac{d[ATP]}{dt} = k_{prod}\frac{M(\mu)}{V(\mu)} - k_g\mu - k_m[ATP] \tag{6}$$

Thus, the steady state ATP concentration can be expressed:

$$[ATP]_{ss} = \frac{k_{prod}}{k_m}\frac{M(\mu)}{V(\mu)} - \frac{k_g}{k_m}\mu \tag{7}$$

M(µ) and V(µ) can be calculated from equations (3-4) using steady state growth rates as measured from this study (Supplementary Table 3).

Supplementary Table 3. Parameters used in the model.

Parameter	Explanation and unit	Glucose	Acetate	Source
μ	Growth rate (h^{-1})	0.59	0.29	[5]
M	Cell mass (10^{-12} gDW/cell)	0.22	0.10	[5]
V	Cell Vol (10^{-15} L/cell)	0.58	0.46	[5]
[ATP]	Intracellular ATP concentration (M)			
$r_{production}$	Intracellular ATP production rate (M/h)			
$r_{consumption}$	Intracellular ATP consumption rate (M/h)			
k_{prod}	ATP production coefficient (ATP moles/gDW/h)			
k_g	ATP consumption coefficient by cell growth (M)			
k_m	Maintenance energy consumption rate coefficient (h^{-1})			

Previous studies treated k_g and k_m as parameters insensitive to carbon-source. When *E. coli* growing at a lower growth rate in acetate than glucose, the second term of Equation (7) contributes to a higher steady state ATP level. We then compare to the first term of Equation (7) between glucose and acetate media by estimating k_{prod}.

Calculating the E. coli ATP production coefficients from carbon metabolism:

When growing in glucose and acetate media, reactions that directly produces and consumes ATP are listed in Supplementary Table 1. Flux through each reaction has been measured by previous studies for *E. coli* growing M9 glucose and acetate media. The net ATP produced from carbon metabolism can thus be calculated from flux as shown in Supplementary Table 4.

Supplementary Table 4. ATP production rate for *E. coli* growing in minimal glucose and acetate media.

	Glucose		Acetate	
**Reactions [a]**	**Flux mmol/(gDW*h)**	**ATP production mmol/(gDW*h)**	**Flux mmol/(gDW*h)**	**ATP production mmol/(gDW*h)**
**Glycolysis**				
Glu + ATP → G6P[b]	7.00	-7.00		
F6P + ATP → FBP[c]	5.67	-5.67		
GAP → 3PG + ATP + NADH[d]	11.69	40.92		

PEP → Pyr +ATP	8.47	8.47		
**PP Pathway**				
G6P → 6PG + NADPH	1.96	4.90	0.70	1.76
6PG → Ru5P + CO2 + NADPH	1.82	4.55	0.70	1.76
**Acetic Acid reactions**				
AcCoA → Ace + ATP	2.31	2.31	-41.40	-41.40
**TCA cycle**				
Pyr → AcCoA + CO2 + NADH	7.07	17.68	0.00	0
ICit → AKG + CO2 + NADPH	1.89	4.73	22.90	57.25
AKG → SucCoA + CO2 + NADH	1.19	2.98	21.50	53.75
SucCoA → Suc + ATP	0.91	0.91	21.50	21.5
Suc → Fum + FADH2	1.33	2.00	30.30	45.45
Mal → OAC + NADH	1.61	4.03	30.30	75.75
**Amphibolic Reactions**				
Mal → Pyr + CO2 + NADH	0.06	0.14	2.70	6.75
OAC +ATP → PEP + CO2	0	0.00	2.70	-2.70
**Net ATP**		80.9		222.6

[a] Only reactions involve in ATP, NAD(P)H, and FADH2 during carbon metabolism are listed.

[b] Assuming all glucose was converted to G6P. Glucose uptake rate was measured from previous experiments using M9 glucose and acetate media, yielding similar growth rates with this study [5].

[c] Flux were calculated using the above glucose uptake rate and flux values from a previous ^{13}C MFA study in M9 glucose medium of similar growth rate [7].

[d] ATP production rate was calculated assuming NAD(P)H → 2.5 ATP, FADH2 → 1.5 ATP [6].

[e] Acetate uptake rate was measured in previous experiments with similar growth rates with this study [8].

Based on Supplementary Table 2, the net ATP production coefficient in acetate k_{prod}^{Ace} is 0.223 mol/(gDW*h), 2.7-fold higher than that in glucose k_{prod}^{Glu}. Taking these values to Equation (2), we obtain $r_{prodction}$ = 31 M/h in glucose and $r_{prodction}$ = 48 M/h in acetate.

For steady state ATP concentration, the first term of Equation (7) is also greater for acetate than that for glucose. Thus, at the steady state, *E. coli* intracellular ATP concentration is higher in acetate than that in glucose due to both a higher ATP production rate (the first term of Equation 7) and a lower consumption rate from its slower growth (the second term of Equation 7).

Supplementary Reference

1. Xiao, Y., Bowen, C.H., Liu, D. & Zhang, F. Exploiting nongenetic cell-to-cell variation for enhanced biosynthesis. *Nat Chem Biol* **12**, 339-344 (2016).
2. Alonso-Gutierrez, J. et al. Metabolic engineering of Escherichia coli for limonene and perillyl alcohol production. *Metab Eng* **19**, 33-41 (2013).
3. Liu, Z.H. et al. Transforming biorefinery designs with 'Plug-In Processes of Lignin' to enable economic waste valorization. *Nat Commun* **12**, 3912 (2021).
4. Cook, T.B. et al. Genetic tools for reliable gene expression and recombineering in Pseudomonas putida. *Journal of industrial microbiology & biotechnology* **45**, 517-527 (2018).
5. Pramanik, J. & Keasling, J.D. Stoichiometric model of Escherichia coli metabolism: Incorporation of growth-rate dependent biomass composition and mechanistic energy requirements. *Biotechnol Bioeng* **56**, 398-421 (1997).
6. Deng, Y., Beahm, D.R., Ionov, S. & Sarpeshkar, R. Measuring and modeling energy and power consumption in living microbial cells with a synthetic ATP reporter. *BMC Biol* **19**, 101 (2021).
7. He, L. et al. Central metabolic responses to the overproduction of fatty acids in Escherichia coli based on 13C-metabolic flux analysis. *Biotechnol Bioeng* **111**, 575-585 (2014).
8. Walsh, K. & Koshland, D.E., Jr. Branch point control by the phosphorylation state of isocitrate dehydrogenase. A quantitative examination of fluxes during a regulatory transition. *The Journal of biological chemistry* **260**, 8430-8437 (1985).
