# Supplementary material for: ATP biosensor reveals microbial energetic dynamics and facilitates bioproduction
Source: Nat Commun. 2024 Jun 21;15:5299. doi: 10.1038/s41467-024-49579-1 (PMC11192931; doi:10.1038/s41467-024-49579-1)
Supplement: Supplementary file 3 — Description of Additional Supplementary Files [file 41467_2024_49579_MOESM3_ESM.pdf]

<u>Description of Additional Supplementary Files</u>

Supplementary Movie 1 - Timelapse video of wildtype E.coli MG1655 cells growing on a agarose pad containing M9 medium with 0.1% glucose.
